# Smoke-free hospitality environments and cognitive health: A population-based study in the United States

**DOI:** 10.1016/j.pmedr.2024.102961

**Published:** 2025-01-03

**Authors:** Lucie Kalousová

**Affiliations:** Departments of Medicine, Health, and Society & Sociology, Vanderbilt University, 2201 West End Ave, Nashville, TN 37235, USA

**Keywords:** Self-rated cognitive decline, AD/RD, Smoke-free laws, Indoor tobacco exposure, Tobacco control, Health disparities, Population health, BRFSS

## Abstract

**Introduction:**

Cigarette smoking is among the largest risk factors for cognitive decline in later life. This study examines the associations between hospitality smoke-free coverage in the US and the prevalence of self-rated cognitive function decline and disparities therein.

**Methods:**

I use the repeated cross-sectional Behavioral Risk Factor Surveillance data collected between 2017 and 2022 from a sample of Americans 45 years and older and estimate logistic regression models predicting self-rated cognitive function decline by calculated smoke-free hospitality coverage in restaurants and bars.

**Results:**

Fully adjusted models indicate a marginally statistically significant 0.16 percentage point reduction [CI −0.35 to 0.02] in the probability of self-rated cognitive function decline for a 10 % increase in the smoke-free bar coverage. The effect is statistically significant and larger for women, a 0.29 [CI −0.50 to −0.01] percentage point decrease, and for non-smokers, a 0.35 [CI −0.56 to −0.15] percentage point decrease. I do not find a parallel effect of smoke-free restaurant laws and I find no effect of either law on self-rated cognitive function decline-related limitations in daily life for either hospitality law.

**Conclusions:**

The findings suggest that smoke-free bar laws could play a role in preventing cognitive decline among older adults in the United States. Effective public health strategies against cognitive decline should include both targeted and broad-based policy measures.

## Highlights

• Cigarette smoke exposure is an important risk factor for later life cognitive decline.
• Smoke-free bar laws associated with a lower probability of cognitive decline.
• Larger effects found for women and non-smokers.
• Public health strategies for cognitive health may benefit from population approaches.

## 1 Introduction

The number of older adults living with mild cognitive impairment and clinical dementia in the United States will double by 2060 (Rajan et al., 2021). Because individuals living with significant cognitive function decline require high-intensity caregiving and support, the anticipated increase has raised concerns among population health scholars, policymakers, and caregivers (Ernst and Hay, 1994). This has led to fast developments in our understanding of the individual health and social characteristics, such as family history or lack of physical activity (Alzheimer's Association, 2019), that contribute to increased cognitive decline risk in later life. However, the existing scholarship has thus far paid much more limited attention to large public health policies that may lower the risk of cognitive decline by acting as protective buffers at the population level. This paper advances current literature by examining the potential of smoke-free hospitality laws to prevent cognitive decline across the U.S. population.

Smoking is among the largest risk factors for cognitive function decline (Orgeta et al., 2019). People who smoke are about 30–50 % more likely to develop Alzheimer's disease (AD) compared to non-smokers and are more likely to report self-rated cognitive function decline (Rajczyk et al., 2023; Durazzo et al., 2014). The adverse effects of smoking on brain health unfold through a variety of pathways, such as increased oxidative stress, inflammation, and vascular damage caused by smoking (Durazzo et al., 2010; Liu et al., 2020). Importantly, people who do not themselves smoke but are exposed to secondhand smoke are also at risk of cognitive function decline through similar physiological mechanisms, and the risk may depend on the duration and intensity of exposure (Chen et al., 2013; Liu et al., 2021). Both smoking prevention and second-hand smoke protection are thus likely to bring about positive effects on cognitive function at the population level.

The share of the U.S. population that smokes has been steadily declining over the last several decades. This trend is expected to contribute to improved population health in the decades to come (Cornelius et al., 2022). The decrease in smoking has been ushered in by strong tobacco control laws and regulations, specifically, tobacco taxes and smoke-free laws (Holford et al., 2014; Chaloupka et al., 2012; DeCicca et al., 2008). Smoke-free laws eliminate opportunities for smoking and help change social norms regarding public smoking (Titus et al., 2019). Smoke-free laws also positively impact non-smokers by shielding them from exposure to secondhand smoke in public spaces (Farrelly et al., 2005). Smoke-free laws have been linked to lower incidences of respiratory (Ho et al., 2017), cardiac (Abreu et al., 2017), and cerebrovascular diseases (Tan and Glantz, 2012) and to improved birth outcomes (Faber et al., 2017). Hospitality laws, that is laws that limit or prohibit smoking in restaurants and bars, are especially powerful because they reduce the prevalence of smoking in public spaces where people typically spend extended periods of time. These laws not only protect workers and patrons but also create a cultural shift toward a smoke-free norm in social settings. Over time, this shift can lead to decreased smoking initiation among youth and contribute to greater success of smokers attempting to quit.

This study builds on the existing literature on the positive population health effects of smoke-free hospitality laws, extending it to examine the potential impact of such laws on the prevalence and distribution of self-rated cognitive decline in the US.

Smoking is highly unequally socially stratified. People with less education, those with lower income, and men are more likely to smoke (Cornelius et al., 2022). Because of the homophilous tendencies of social networks (McPherson et al., 2001), the unequal rates of smoking in the population lead to greater rates of secondhand smoke exposure among people from these groups even if they do not smoke. By curbing secondhand tobacco exposure for all groups, smoke-free laws could help eliminate the exposure inequalities in social settings. The second goal of this study is to investigate whether smoke-free hospitality laws contribute to lowering disparities in cognitive function decline by education, sex, and smoking status.

This study uses a large national dataset to ask two related research questions: First, has the adoption of smoke-free hospitality laws, specifically, restaurant and bar laws, been associated with a decreased probability of self-rated cognitive function decline in residents of states with higher protective coverage? Second, has the adoption of smoke-free laws contributed to narrowing the disparities in self-rated cognitive function decline by education, sex, and smoking status? I conclude the paper with a discussion of smoke-free laws as a population health strategy for preventing cognitive function decline.

## 2 Methods

### 2.1 Data

This study employs data from the Behavioral Risk Factor Surveillance System (BRFSS). (Centers for Disease Control and Prevention, 2023) The BRFSS is an annual telephone survey conducted by state health departments with support and oversight from the Centers for Disease Control and Prevention (CDC) and interviews non-institutionalized US residents 18 years of age and older. The survey comprises three primary elements: a set of core questions that are uniformly asked across all states, optional modules introduced by the CDC that are adopted by states on a voluntary basis, and state-specific questions. The Self-rated Cognitive Decline (SCD) Module is optional for states (although all states have administered the module at least once) and investigates recent memory and self-rated cognitive changes among individuals aged 45 and older (Lee and Moore, 2020). This analysis utilizes the most recent six years of available data, spanning from 2017 to 2022, during which 39 US states administered the SCD module. The BRFSS-created survey weights were used to account for complex sampling and clustering of respondents at each sampling level, including state of residence. The study uses de-identified data and is not classified as human subject's research. It is exempt from Institutional Review Board oversight and ethics approval was not required.

### 2.2 Self-rated cognitive decline

Age-eligible respondents were asked if they “experienced confusion or memory loss that is happening more often or is getting worse” during the last 12 months. Those who agreed were classified as having SCD (10 %).

### 2.3 Self-rated cognitive decline limitations

Respondents who reported SCD were subsequently asked about life domains in which confusion or memory loss could present hurdles, including housework activities and chores, work, or social activities. A respondent who responded confirming that SCD creates obstacles to engaging in at least one of these domains was classified as having SCD with limitations (69 % of those with SCD).

### 2.4 Smoke-free hospitality laws coverage

I estimated the proportion of the population protected by comprehensive smoke-free hospitality regulations using the American Nonsmokers' Rights Foundation (ANRF) Tobacco Control Laws Database (American Nonsmokers' Rights Foundation, 2018). Only regulations that fulfilled the ANRF's definition of “100 percent smoke-free” were included (100 Smokefree Definitions. American Nonsmokers' Rights Foundation, 2018). I merged information on smoke-free ordinances enacted at various governmental levels—city, county, and state—with intercensal and census data (Gonzalez et al., 2014). This allowed me to calculate the population-weighted fraction of each state that was covered by each type of law by year. This method adjusts for differences in population density, ensuring that areas with larger populations are appropriately represented. However, it does not provide an accurate measure of individual-level exposure in specific local contexts.

Because I anticipate that smoke-free coverage is likely to have cumulative positive effect on outcomes, I constructed three measures of cumulative coverage. The first captured average coverage over five years, the second over ten years, and the third over twenty years. The main results presented in this paper show the findings for the twenty-year average coverage models; the results for five- and ten-year average coverage models are provided as supplemental materials (Supplementary Figs. 1A and 1B).

Available literature on the physiological mechanism by which tobacco smoke affects brain health points to gradual effects (Durazzo et al., 2010; Csabai et al., 2016). I therefore also anticipate that protective effects of smoke-free coverage may only be realized several years after implementation. To allow for this, I lagged the exposure to smoke-free laws by five years. In practice, this means that the exposure variable for a person living in, for example, Texas in 2021, would be 21 % coverage for restaurant laws and 15 % for bar laws, which represent the twenty-year average protection in each domain from 1996 to 2016 for the state's residents. In sensitivity analysis, I evaluated alternative lag durations of three and seven years. The results were not substantively different.

### 2.5 Control variables

The regression models control for several additional important factors, which may explain the relationship between smoke-free hospitality laws and SCD. These include a binary indicator for sex (male vs. female), categorical indicators for race and ethnicity (White non-Hispanic, Black non-Hispanic, Hispanic, or member of another non-Hispanic group; imputed by the BRFSS survey team for respondents who did not provide a value), marital status (married or partnered, formerly partnered, or never married), education (less than high school, high school graduate, some college, and college or higher), household income (less than $25,000, $25,000 to 49,999, $50,000 to 74,999, and $75,000 or more), and smoking status (never a smoker, current smoker, and former smoker), and indicators for whether a respondent has ever had heart disease (no/yes), stroke (no/yes), or felt sad or depressed during any of the last 30 days (no/yes). In addition, the final set of regression models controls for the year of data collection, U.S. census region, and average cigarette tax over the last twenty years in the state. Cigarette tax exposure was calculated using the Tax Burden of Tobacco dataset (Orzechowski and Walker, 2023) and adjusted for inflation to the 2022 consumer price index to maintain comparability across years.

### 2.6 Analytic samples

The first analytic sample consisted of all age-eligible respondents (initial *n* = 236,501), who provided the data necessary for the construction of the SCD outcome variable and control variables. The largest source of missing data was the income variable, with approximately 18 % missing (43,232), followed by the number of days depressed (5292), SCD (2366), and heart disease (2675). For all remaining variables, missingness was less than 2000. After listwise deletion, the first analytic sample had184,203 respondents. The second analytic sample was a subset of the first, including only respondents who indicated SCD. I exclude responses from those who did not provide answers to questions regarding SCD-related limitations (518). The final sample size for the second analytic sample was 18,427 respondents.

### 2.7 Statistical analysis

**Fig. 1:**
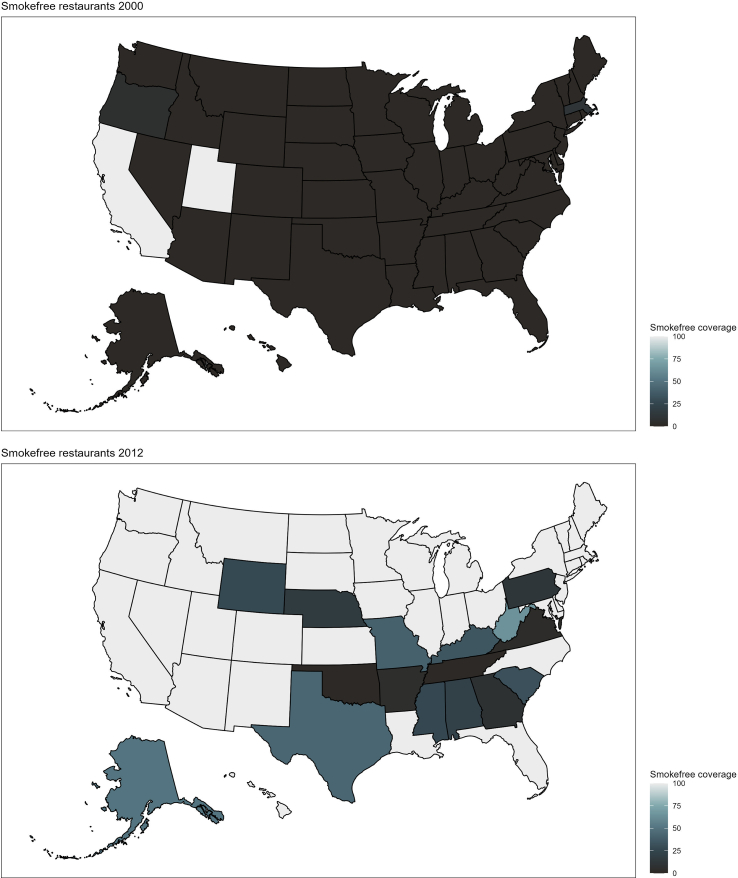

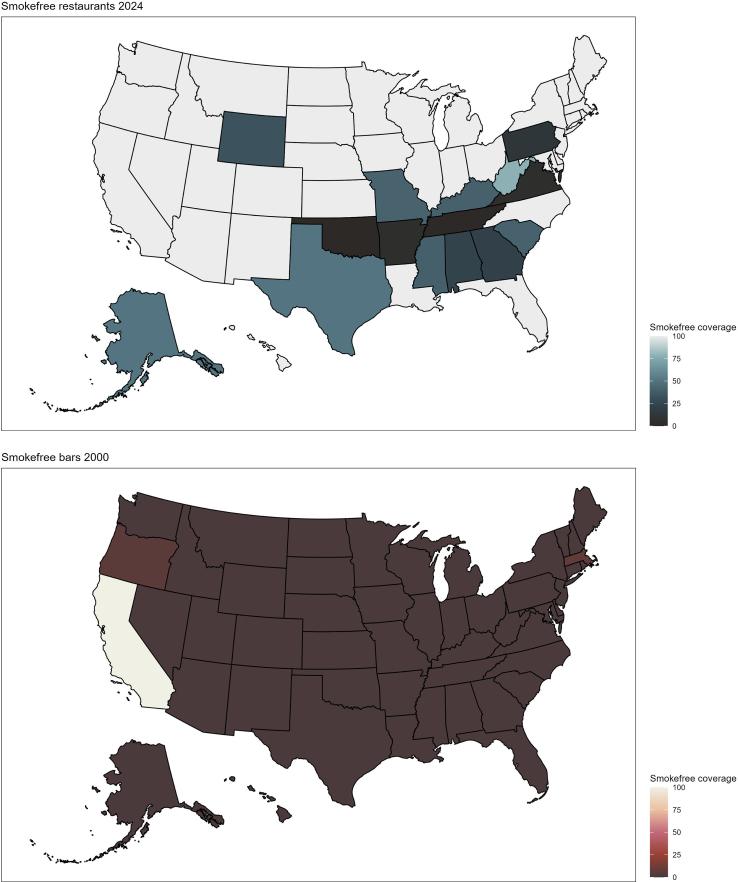

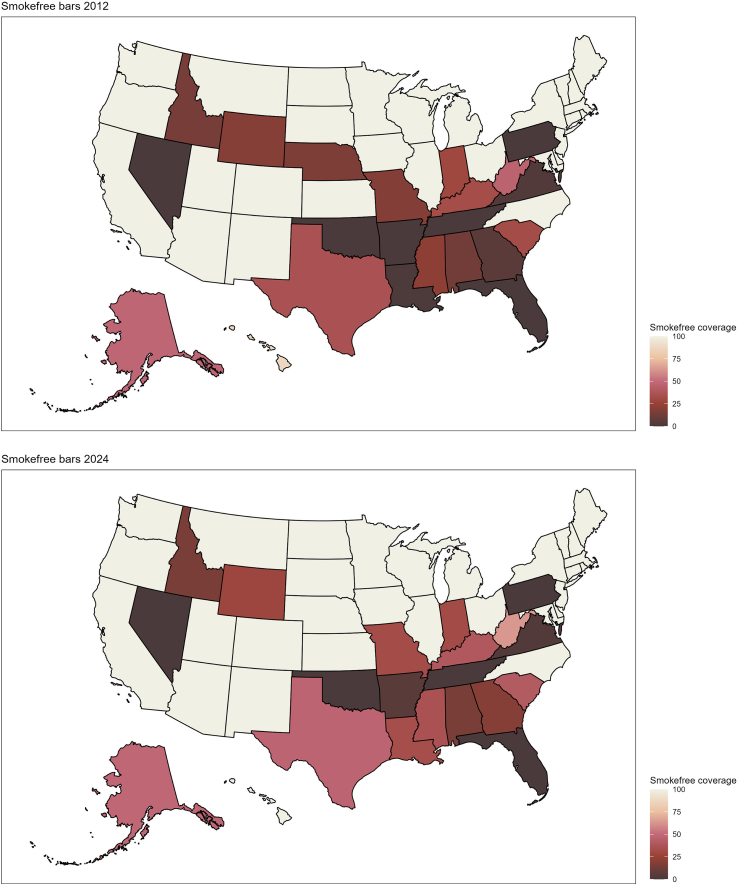
The adoption of smoke-free restaurant and bar coverage in the United States over time pictured in 2000, 2012, and 2024.

**Fig. 2:**
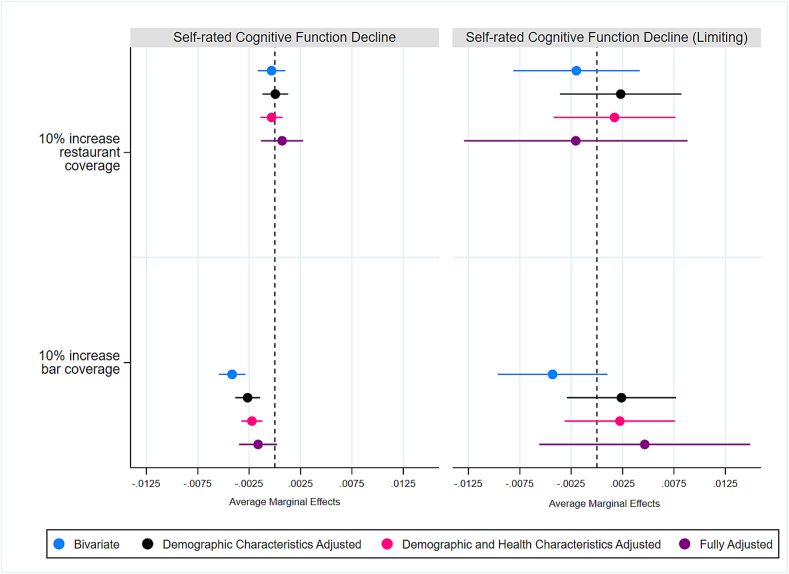
Average marginal effects and 95 % confidence intervals from logistic regression models predicting self-rated cognitive function decline by restaurant and bar smoke-free coverage over the last twenty years with five-year lags in the Behavioral Risk Factor Surveillance System analytic sample collected between 2017 and 2022 in the United States. *Note*: Survey weights applied. Covariates held at means. Demographic characteristics adjusted models control for sex, race/ethnicity, education, income, marital status and age. Demographic and health characteristics adjusted models add controls for heart disease, stroke, days depressed, and current smoking status. Fully adjusted models add controls for census region, state cigarette taxes, and year of data collection.

**Fig. 3:**
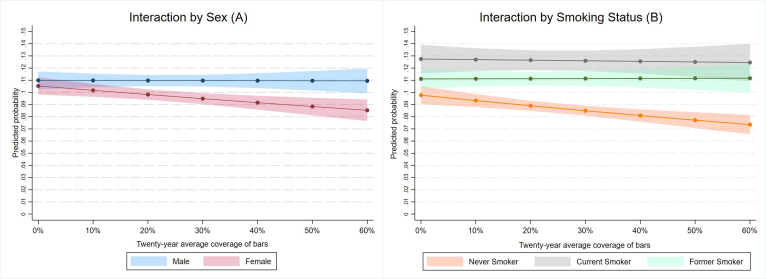
Predicted probability and 95 % confidence intervals of self-rated cognitive function decline estimated by fully adjusted logistic regression models in the Behavioral Risk Factor Surveillance System analytic sample collected between 2017 and 2022 in the United States. *Note*: Survey weights applied. Covariates held at means. Fully adjusted models control for sex, race/ethnicity, education, income, marital status, age, heart disease, stroke, days depressed, current smoking status, for census region, state cigarette taxes, and year of data collection.

Fig. 1 shows how the share of the U.S. population protected by smoke-free hospitality laws developed between the years 2000 and 2024. I begin analysis by examining the descriptive characteristics for the overall sample and those with and without SCD. The results are presented in Table 1. Then, I estimate logistic regression models to predict the likelihood of SCD and SCD with limitations using the percent of the state population covered by smoke-free restaurant or bar laws as predictors. The results are visually presented in Fig. 2 and full models are included in Supplementary Table 1. The associations are presented first unadjusted, then I adjust for demographic characteristics (sex, race, education, income, marital status, and age); in the third set, I add controls for health (heart disease, stroke, depression, and smoking status); and in the final set of fully adjusted models, I add controls for area characteristics (census region, average cigarette tax) and year fixed effects. Finally, I estimate whether the effects of the laws were modified by respondent characteristics using the fully adjusted logistic regression models specifications with interactions between each type of smoke-free coverage and respondent characteristics (sex, education, smoking status). The results from statistically significant interactions are shown in Fig. 3 and both statistically significant and non-significant interactions are shown in Supplementary Table 2. To facilitate cross-model comparison, all results are presented as average marginal effects (AME's) or predicted probabilities.

**Table 1:** Descriptive characteristics of the Behavioral Risk Factor Surveillance System analytic sample overall and stratified by self-rated cognitive function decline collected between 2017 and 2022 in the United States.

	Overall		No SCD		Yes SCD		p for diff
	Svy. Mean or %	Svy. SE (Mean)	Svy. Mean or %	Svy. SE (Mean)	Svy. Mean or %	Svy. SE (Mean)	
Self-rated cognitive decline in the last year	10	–	–	–	–	–	
Limitations due to self-rated cognitive decline	–	–	–	–	69	–	
Average smoke-free restaurant coverage	26	–	26	–	26	–	0.635
Average smoke-free bar coverage	18	–	18	–	16	–	<0.001
Average cigarette taxes in the last twenty years	1.20	(0.00)	1.20	(0.00)	1.15	(0.01)	<0.001
Female	52	–	52	–	54	–	0.023
Race/ethnicity							
White Non-Hispanic	74	–	74	–	72	–	0.291
Black Non-Hispanic	12	–	12	–	12	–	
Hispanic	9	–	9	–	10	–	
Other Non-Hispanic	5	–	5	–	5	–	
Marital Status							
Married/partnered	63	–	65	–	52	–	<0.001
Formerly partnered	29	–	28	–	39	–	
Never married	8	–	8	–	10	–	
Education							
Less than high school	12	–	11	–	19	–	<0.001
High school graduate	28	–	27	–	31	–	
Some college	31	–	31	–	30	–	
College+	30	–	31	–	20	–	
Household income last year							
$0 to $24,999	24	–	22	–	42	–	<0.001
$25,000 to $49,999	23	–	23	–	26	–	
$50,000 to $74,999	16	–	16	–	12	–	
$75,000+	37	–	39	–	20	–	
Smoking status							
Never smoker	54	–	55	–	41	–	<0.001
Current smoker	15	–	14	–	24	–	
Former smoker	32	–	31	–	36	–	
Ever Had Heart Disease	7	–	6	–	13	–	<0.001
Ever Had a Stroke	6	–	5	–	13	–	<0.001
Any days depressed in the past 30 days	31	–	27	–	62	–	<0.001
Age	61.76	(0.05)	61.64	(0.06)	62.81	(0.20)	
N	184,203		165,258		18,945		

*Note:* Survey weights applied. Wald test used for calculating the *p*-values of differences between means. Chi-square test used for calculating the *p*-values of differences between percentages.

## 3 Results

Fig. 1 shows the percentage of the population in each state that is covered by comprehensive smoke-free restaurant or bar laws enacted at either local or state levels. The first state to adopt a 100 % smoke-free restaurant law was Utah in 1995, followed by California, which adopted both smoke-free restaurants and bars in 1998. In 2024, about 67 % of the U.S. population lived in areas covered by comprehensive protection in both restaurants and bars.

Table 1 shows the descriptive characteristics for the sample. Respondents with and without SCD had similar levels of smoke-free restaurant coverage (26 %). However, I find greater smoke-free bar coverage (18 vs. 16 %) and a higher average cigarette tax burden ($1.20 [1.20–1.21] vs. $1.15 [1.14–1.17]) among respondents without SCD. Women comprised a greater share of those with SCD (54 %). People who were married were underrepresented among those with SCD compared to those without (52 vs. 65 %), and I find large differences in educational attainment between the two groups. The group without SCD included a smaller share of people who had less than a high school degree (11 vs. 19 %) and a larger share of people with a college degree or higher (31 vs. 20 %). I also find that the group without SCD had a greater share of people who were never smokers (55 vs. 41 %) and a lower share of current smokers (14 vs. 24 %). Fewer people without SCD reported having had heart disease (6 vs. 13 %) or a stroke (5 vs. 13 %), and they described themselves as being sad or depressed on any recent day less often (27 vs. 62 %). The group without SCD was also on average younger (61.6 [61.53–61.75] vs. 62.8 [62.42–63.20] years).

Fig. 2 shows the results from the logistic regression models predicting changes in the probability of SCD by smoke-free restaurant and bar coverage. The first set of models shows bivariate associations, the second adjusts for demographic characteristics, the third adds controls for health, and the final set adds controls for census region, average cigarette tax and year fixed effects. I find no statistically significant associations between restaurant smoke-free coverage and either SCD or SCD-related limitations. In contrast, greater smoke-free coverage in bars is associated with a lower probability of SCD. A ten-percentage-point increase in smoke-free bar coverage is associated with a 0.4 [−0.55 to −0.29] percentage point reduction in the probability of SCD in the bivariate models, a 0.3 [−0.39 to −0.14] percentage point reduction in the models adjusted for demographic characteristics, a 0.2 [−0.33 to −0.12] percentage point reduction in the models adjusted for demographic and health characteristics, and a 0.2 [−0.35 to 0.02] percentage point reduction in the fully adjusted models. The AMEs are statistically significantly different from zero in all four models in the set that predicts SCD by smoke-free bar coverage, although the association is only marginally statistically significant in the fully adjusted model. The associations in any models predicting SCD with limitations are not statistically significant.

Fig. 3A and B show results of the statistically significant interactions predicting SCD by smoke-free bar coverage in the fully adjusted models. Fig. 3A shows that the probability of SCD was attenuated more substantially for female respondents when smoke-free bar laws are implemented. While male and female respondents had similar probability of SCD when the average smoke-free coverage was none or close to zero, female respondents' probability of SCD was significantly lower in areas with greater average coverage.

Fig. 3B shows that the probability of SCD was lower for non-smokers regardless of the extent of smoke-free bar coverage. The difference was greater in areas with smoke-free bar coverage. In areas with no or very little smoke-free bar coverage, the probability of SCD among non-smokers was 1.3 [−2.28 to −0.39] percentage points lower than among former smokers and 2.9 [−4.23 to −1.71] percentage points lower than among current smokers. In areas where coverage reached 60 %, the probability of SCD among non-smokers was 3.4 [−4.32 to −2.56] percentage points lower than among former smokers and 4.8 [−6.02 to −3.55] percentage points lower than among current smokers. I also found suggestive evidence of the bar laws' greater positive effects on people with more education. All groups with more than high school showed a marginally statistically significant decline in areas with greater coverage, however, the interaction overall was not statistically significant. The AME's from these and the remainder of the models, including those that did not yield statistically significant interactions, are available in Supplemental Table 2.

## 4 Limitations

The use of a large comprehensive dataset is a strength of this study. However, the repeated cross-sectional design does not allow us to track individual changes in SCD over time. This study shows variation in the risk of SCD across different smoke-free exposures measured at the aggregate state level, rather than dynamic within-individual changes. There may be inaccuracies in assessing smoke-free exposure for individuals who have relocated across states within the past several decades. About 2.3 % of the U.S. population moves between states annually (U.S. Census Bureau, 2023), and this could lead to potentially inaccurate measurement of exposure and duration of exposure to smoke-free laws for those who have moved. The likelihood of such a measurement error is somewhat mitigated by the older average age of the people who were included in the BRFSS SCD module because interstate relocations are less common later in life (Frost, 2020).

This study measures smoke-free exposure by the percentage of a state's population that has been protected by hospitality smoke-free laws over time. Smoke-free laws are sometimes passed by very small geopolitical units, e.g., municipalities, which the measures capture, but which I am not able to spatially link to individual respondents. The smoke-free coverage measure is thus an approximation of the degree to which a resident of a state is covered. If a significant share of the population were to travel to neighboring states without smoke-free regulations (or the reverse), the coverage estimates would not capture the true exposure to smoke-free environments for the residents of a state. Research on early smoke-free hospitality laws found no significant shifts in consumer behavior after their enactment and does not support the hypothesis that cross-boundary travel to avoid or seek out smoke-free establishments is widespread (Loomis et al., 2013). Future research should explore calculating coverage at lower geographic levels and linking individual respondents to specific coverage level using access restricted geographic data. The severity of this issue is somewhat diminished by the policy level at which most people are protected. During the last two decades, most gains in coverage have been made by comprehensive state-level laws, effectively eliminating local variation in many states. Although residents of some states are only covered by a patchwork of local laws, most people covered by smoke-free laws are covered under state-level coverage or both state-level and local coverage.

Finally, while the use of SCD as an outcome measure—consistently employed by the CDC since 2010—is among the study's strengths, it does not match the diagnostic precision of clinical evaluations. A recent meta-analysis of prospective longitudinal studies shows that individuals with SCD are over two times more likely to transition to dementia and mild cognitive impairment than those without SCD (Parfenov et al., 2020) but this self-rated indicator might not fully encompass the experiences of those individuals whose cognitive impairments are apparent to their friends and family but remain unrecognized by the individuals themselves.

## 5 Discussion

This study investigated whether the benefits that smoke-free laws could be effective as a policy tool for preventing cognitive function decline and how they might contribute to or mitigate previously documented disparities in cognitive outcomes. The findings show that people who live in areas with a greater proportion of population protected by smoke-free bars have a lower probability of SCD. I found no difference in the likelihood they will experience SCD-related limitations. These results suggest that while smoke-free bar laws might have an impact on preventing the transition to self-rated cognitive decline, they may not influence the speed or the severity of the symptoms.

I find no parallel results for smoke-free restaurant laws. This may be due to several reasons. Opinion surveys report that people are less likely to tolerate smoking in restaurants than in bars (Saad, 2010). This suggests that secondhand smoke exposure is already less common in restaurants, and restaurant laws consequently reduce exposure less. An alternative explanation could be that smoke-free bar laws are a stronger proxy for an active tobacco control policy within a state or local area. Because fewer people believe smoking should be banned in bars (Gallup News Service, 2014), a smoke-free bar law could indicate a stronger broad political support for a broad range of tobacco control measures, and this combination could translate to better brain health at the population level.

I find a larger positive effect on women and non-smokers. These findings align with the expectation based on the laws' protective intent. People who do not smoke are less likely to be exposed to secondhand smoke after the laws' implementation, and smoke-free laws most enhance the health advantage of such individuals. Women, too, disproportionately benefit from smoke-free coverage. Prior research shows that smoking patterns are gendered. Women smoke fewer cigarettes on average (Peters et al., 2014) and may attach a greater meaning to social smoking (Paul et al., 2010). Smoke-free bar laws may thus have a greater impact on reducing women's secondhand smoke exposure compared to men's. Overall, the findings suggest that smoke-free laws may have a mixed effect on SCD disparties, potentially decreasing gender disparities and enhancing the advantage of non-smokers.

## 6 Conclusions

As the population prevalence of cognitive decline among older U.S. adults rises, it becomes increasingly urgent to identify policies and large-scale interventions that may protect brain health at the population level and prevent negative outcomes in later life. This study provides preliminary evidence supporting the hypothesis that smoke-free bar laws may be one such promising population health policy. These findings underscore the importance of smoke-free bar laws as a public health strategy to prevent cognitive decline, particularly among non-smokers and women. For people who do not smoke but spend time or work in bars, the gains could be substantial. The findings align with other research that has identified smoke-free laws' benefits for cardiovascular and pulmonary health (Hahn et al., 2011; Menzies et al., 2006), among others. Future research should continue exploring macrosocial policy settings that shape risks at the community and state levels (Galea and Putnam, 2007), building on the preliminary evidence provided here.

## Funding

This work was supported by an 10.13039/100000957Alzheimer's Association Grant (AARG-D-NTF-22-924330).

## CRediT authorship contribution statement

**Lucie Kalousová:** Writing – review & editing, Writing – original draft, Visualization, Validation, Software, Resources, Project administration, Methodology, Investigation, Funding acquisition, Formal analysis, Data curation, Conceptualization.

## Declaration of competing interest

The author declares that they have no known competing financial interests or personal relationships that could have appeared to influence the work reported in this paper.

## Data Availability

The authors do not have permission to share data.

## Appendix A Supplementary data

Supplementary tables and figures Image 1

## References

[bb0005] 100 Smokefree Definitions. American Nonsmokers’ Rights Foundation (2024). https://no-smoke.org/100-smokefree-definitions/.

[bb0010] Abreu D., Sousa P., Matias-Dias C., Pinto F.J. (2017). Longitudinal impact of the smoking ban legislation in acute coronary syndrome admissions. Biomed. Res. Int..

[bb0015] Alzheimer's Association (2019). 2019 Alzheimer's disease facts and figures. Alzheimers Dement..

[bb0020] American Nonsmokers' Rights Foundation (2018). U.S. Tobacco Control Laws Database. https://no-smoke.org/materials-services/lists-maps/.

[bb0035] Centers for Disease Control and Prevention (2023). Behavioral Risk Factor Surveillance System Survey Data. https://www.cdc.gov/brfss/annual_data/annual_data.htm.

[bb0040] Chaloupka F.J., Yurekli A., Fong G.T. (2012). Tobacco taxes as a tobacco control strategy. Tob. Control..

[bb0045] Chen R., Hu Z., Orton S., Chen R.L., Wei L. (2013). Association of passive smoking with cognitive impairment in nonsmoking older adults: a systematic literature review and a new study of Chinese cohort. J. Geriatr. Psychiatry Neurol..

[bb0050] Cornelius M.E., Loretan C.G., Wang T.W., Jamal A., Homa D.M. (2022). Tobacco product use among adults: United States, 2020. MMWR Morb. Mortal Wkly. Rep..

[bb0055] Csabai D., Csekő K., Szaiff L. (2016). Low intensity, long term exposure to tobacco smoke inhibits hippocampal neurogenesis in adult mice. Behav. Brain Res..

[bb0060] DeCicca P., Kenkel D., Mathios A. (2008). Cigarette taxes and the transition from youth to adult smoking: smoking initiation, cessation, and participation. J. Health Econ..

[bb0065] Durazzo T.C., Meyerhoff D.J., Nixon S.J. (2010). Chronic cigarette smoking: implications for neurocognition and brain neurobiology. Int. J. Environ. Res. Public Health.

[bb0070] Durazzo T.C., Mattsson N., Weiner M.W. (2014). Smoking and increased Alzheimer’s disease risk: a review of potential mechanisms. Alzheimers Dement..

[bb0075] Ernst R.L., Hay J.W. (1994). The US economic and social costs of Alzheimer’s disease revisited. Am. J. Public Health.

[bb0080] Faber T., Kumar A., Mackenbach J.P. (2017). Effect of tobacco control policies on perinatal and child health: a systematic review and meta-analysis. Lancet Public Health.

[bb0085] Farrelly M.C., Nonnemaker J.M., Chou R., Hyland A., Peterson K.K., Bauer U.E. (2005). Changes in hospitality workers’ exposure to secondhand smoke following the implementation of New York’s smoke-free law. Tob. Control..

[bb0090] Frost R. (2020). Are Americans stuck in place? declining residential mobility in the US. https://www.jchs.harvard.edu/blog/who-is-moving-and-why-seven-questions-about-residential-mobility.

[bb0095] Galea S., Putnam S. (2007). Macrosocial Determinants of Population Health.

[bb0100] Gonzalez M., Sanders-Jackson A., Glantz S.A. (2014). Association of strong smoke-free laws with dentists’ advice to quit smoking, 2006–2007. Am. J. Public Health.

[bb0105] Hahn E.J., Rayens M.K., Burkhart P.V., Moser D.K. (2011). Smoke-free laws, gender, and reduction in hospitalizations for acute myocardial infarction. Public Health Rep..

[bb0110] Ho V., Ross J.S., Steiner C.A. (2017). A nationwide assessment of the association of smoking bans and cigarette taxes with hospitalizations for acute myocardial infarction, heart failure, and pneumonia. Med. Care Res. Rev..

[bb0115] Holford T.R., Meza R., Warner K.E. (2014). Tobacco control and the reduction in smoking-related premature deaths in the United States, 1964–2012. JAMA.

[bb0125] Lee S.H., Moore L. (2020). BRFSS statistical brief: cognitive decline optional module. https://www.cdc.gov/aging/data/BRFSS-statistical-brief-cognitive-decline-508.pdf.

[bb0130] Liu Y., Li H., Wang J. (2020). Association of cigarette smoking with cerebrospinal fluid biomarkers of neurodegeneration, neuroinflammation, and oxidation. JAMA Netw. Open.

[bb0135] Liu W., Wang B., Xiao Y., Wang D., Chen W. (2021). Secondhand smoking and neurological disease: a meta-analysis of cohort studies. Rev. Environ. Health.

[bb0140] Loomis B.R., Shafer P.R., van Hasselt M. (2013). The economic impact of smoke-free laws on restaurants and bars in 9 States. Prev. Chronic Dis..

[bb0145] McPherson M., Smith-Lovin L., Cook J.M. (2001). Birds of a feather: homophily in social networks. Annu. Rev. Sociol..

[bb0150] Menzies D., Nair A., Williamson P.A. (2006). Respiratory symptoms, pulmonary function, and markers of inflammation among bar workers before and after a legislative ban on smoking in public places. JAMA.

[bb0155] Orgeta V., Mukadam N., Sommerlad A., Livingston G. (2019). The Lancet Commission on Dementia Prevention, Intervention, and Care: a call for action. Ir. J. Psychol. Med..

[bb0160] Orzechowski and Walker (2023). The tax burden of tobacco. https://chronicdata.cdc.gov/d/fip8-rcng.

[bb0165] Parfenov V.A., Zakharov V.V., Kabaeva A.R., Vakhnina N.V. (2020). Self-rated cognitive decline as a predictor of future cognitive decline: a systematic review. Dement Neuropsychol..

[bb0170] Paul C.L., Ross S., Bryant J., Hill W., Bonevski B., Keevy N. (2010). The social context of smoking: a qualitative study comparing smokers of high versus low socioeconomic position. BMC Public Health.

[bb0175] Peters S.A., Huxley R.R., Woodward M. (2014). Do smoking habits differ between women and men in contemporary western populations? Evidence from half a million people in the UK Biobank study. BMJ Open.

[bb0180] Rajan K.B., Weuve J., Barnes L.L., McAninch E.A., Wilson R.S., Evans D.A. (2021). Population estimate of people with clinical Alzheimer’s disease and mild cognitive impairment in the United States (2020–2060). Alzheimers Dement..

[bb0185] Rajczyk J.I., Ferketich A., Wing J.J. (2023). Relation Between Smoking Status and Self-rated Cognitive Decline in Middle Age and Older Adults: A Cross-Sectional Analysis of 2019 Behavioral Risk Factor Surveillance System Data. J. Alzheimers Dis..

[bb0190] Saad L. (2010). Americans want smoking off the menu at restaurants; more than half believe workplaces and hotels should accommodate smokers. Gallup Poll News Service.

[bb0195] Gallup Organization (2014). Tobacco and smoking. http://www.gallup.com/poll/1717/tobacco-smoking.aspx.

[bb0200] Tan C.E., Glantz S.A. (2012). Association between smoke-free legislation and hospitalizations for cardiac, cerebrovascular, and respiratory diseases: a meta-analysis. Circulation.

[bb0205] Titus A.R., Kalousova L., Meza R. (2019). Smoke-free policies and smoking cessation in the United States, 2003–2015. Int. J. Environ. Res. Public Health.

[bb0210] U.S. Census Bureau (2023).

