# Supplementary material for: Smoke-free hospitality environments and cognitive health: A population-based study in the United States
Source: Prev Med Rep. 2025 Jan 3;50:102961. doi: 10.1016/j.pmedr.2024.102961 (PMC11770490; doi:10.1016/j.pmedr.2024.102961)
Supplement: Supplementary file 1 — Supplementary tables and figures [file mmc1.docx]

**Supplementary Figures 1A and 1B.** Average marginal effects and 95% confidence intervals from logistic regression models estimated in the BRFSS analytic sample predicting SCD and SCD with limitations by average smoke-free restaurant and bar exposure over the last five and ten years with five-year lags.

**Supplementary Figure 1A: Average five-year exposure**


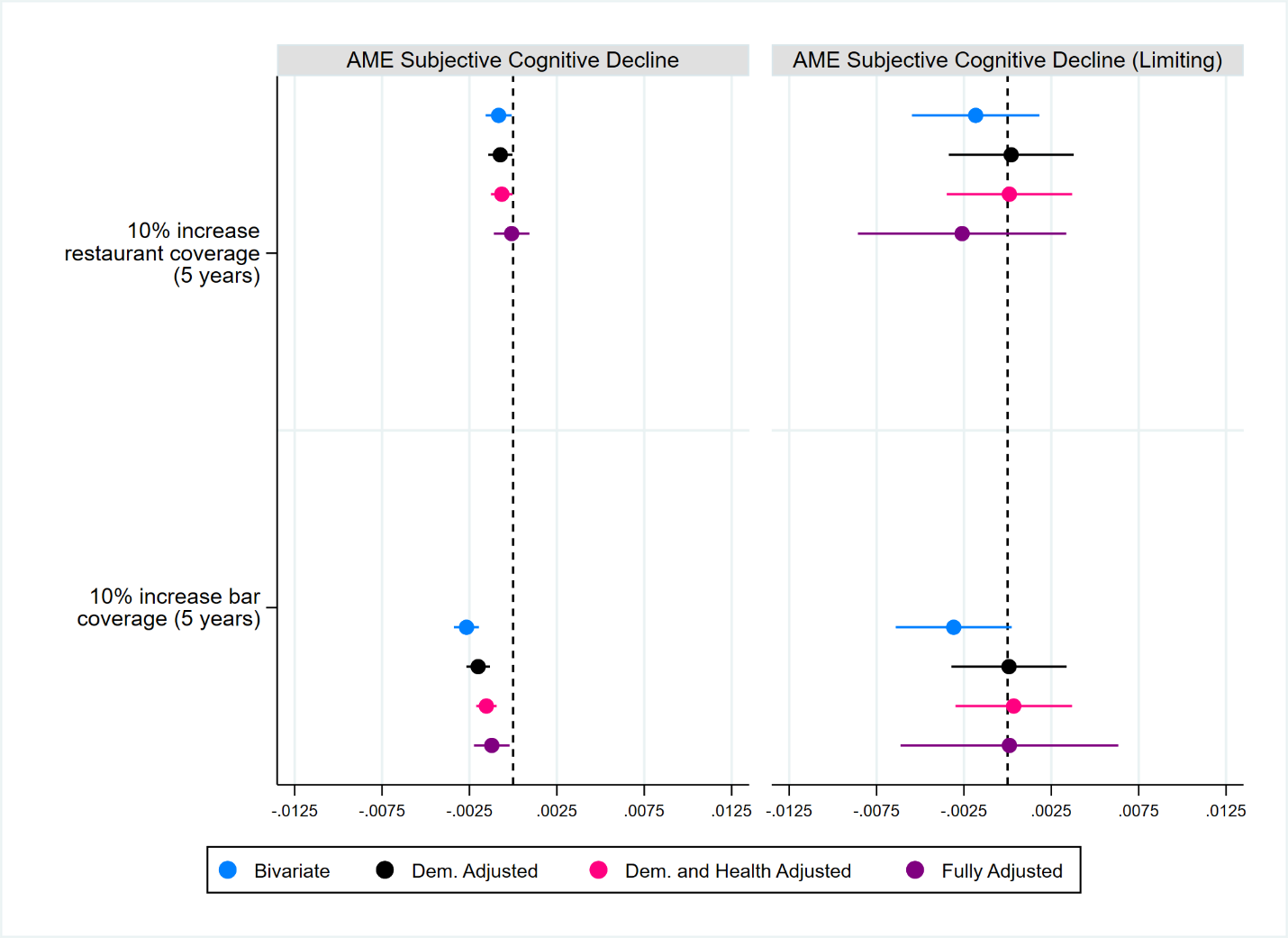


**Supplementary Figure 1B: Average ten-year exposure**


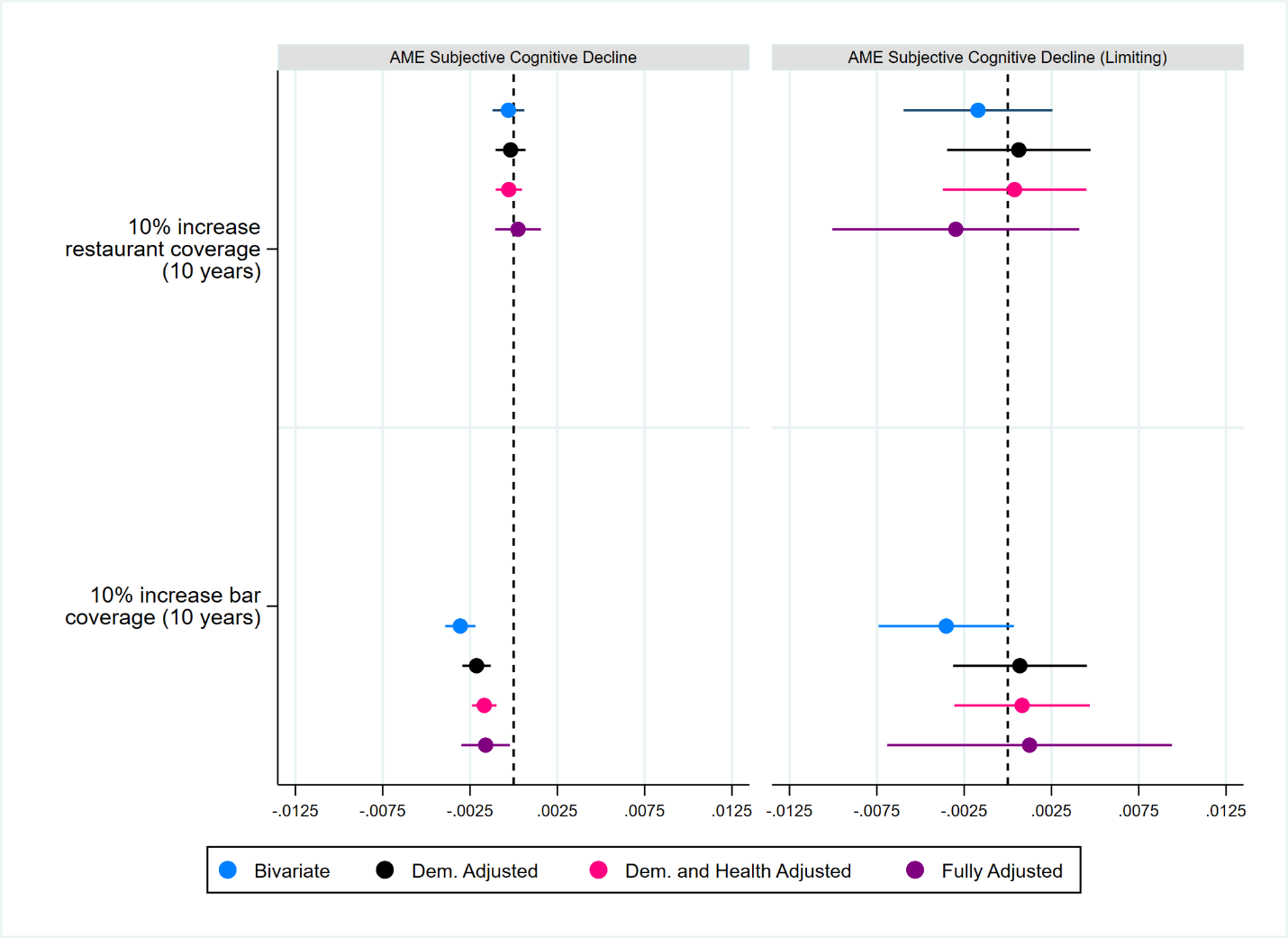


*Note*: Survey weights applied. Covariates held at means. Demographic characteristics adjusted models control for sex, race/ethnicity, education, income, marital status and age. Demographic and health characteristics adjusted models add controls for heart disease, stroke, days depressed, and current smoking status. Fully adjusted models add controls for census region, state tobacco cigarette taxes, and year of data collection.

**Supplementary Table 1.** Average marginal effects and standard errors from logistic regression models estimated in the BRFSS analytic sample predicting SCD and SCD with limitations by 10% increase in average smoke-free restaurant and bar exposure over the last twenty years with five-year lags Covariates held at means. Survey weights applied. Displayed also as Figure 2 in main results.

|  |  | **Subjective Cognitive Decline (SCD)** | | | | | | | |
| --- | --- | --- | --- | --- | --- | --- | --- | --- | --- |
|  |  | Model 1 | Model 2 | Model 3 | Model 4 | Model 1 | Model 2 | Model 3 | Model 4 |
|  |  | AME/SE | AME/SE | AME/SE | AME/SE | AME/SE | AME/SE | AME/SE | AME/SE |
| 10% increase in restaurant coverage over the last twenty years | | -.0003 | .0000 | -.0003 | .0007 |  |  |  |  |
|  |  | (.0007) | (.0006) | (.0006) | (.0010) |  |  |  |  |
| 10% increase in bar coverage over the last twenty years | |  |  |  |  | -0.0042*** | -0.0026*** | -0.0022*** | -0.0016+ |
|  |  |  |  |  |  | (0.0007) | (0.0006) | (0.0005) | (0.0009) |
| n |  | 184,203 | 184,203 | 184,203 | 184,203 | 184,203 | 184,203 | 184,203 | 184,203 |
|  |  |  |  |  |  |  |  |  |  |
|  |  | **Subjective Cognitive Decline (SCD) with Limitations** | | | | | | | |
|  |  | Model 1 | Model 2 | Model 3 | Model 4 | Model 1 | Model 2 | Model 3 | Model 4 |
|  |  | AME/SE | AME/SE | AME/SE | AME/SE | AME/SE | AME/SE | AME/SE | AME/SE |
| 10% increase in restaurant coverage over the last twenty years | | -0.0020 | 0.0023 | 0.0017 | -0.0021 |  |  |  |  |
|  |  | (0.0031) | (0.0030) | (0.0030) | (0.0055) |  |  |  |  |
| 10% increase in bar coverage over the last twenty years | |  |  |  |  | -0.0043 | 0.0024 | 0.0022 | 0.0047 |
|  |  |  |  |  |  | (0.0027) | (0.0027) | (0.0027) | (0.0052) |
| n |  | 18,427 | 18,427 | 18,427 | 18,427 | 18,427 | 18,427 | 18,427 | 18,427 |

+p< 0.1; *p<0.05; **p<0.01; ***p<0.001.

**Supplementary Table 2.** Average marginal effects and standard errors from logistic regression models with interactions estimated in the BRFSS analytic sample predicting SCD and SCD with limitations by 10% increase in smoke-free bar exposure. Covariates held at means. Survey weights applied. +p< 0.1; *p<0.05; **p<0.01; ***p<0.001. Also displayed in Figures 3A and 3B in main results.

|  | Subjective Cognitive Decline (SCD) | | | | | | Subjective Cognitive Decline (SCD) with Limitations | | | | | |
| --- | --- | --- | --- | --- | --- | --- | --- | --- | --- | --- | --- | --- |
|  | Restaurant Laws | | |  | Bar Laws |  | Restaurant Laws | | | Bar Laws | | |
|  | Model 1 | Model 2 | Model 3 | Model 4 | Model 5 | Model 6 | Model 1 | Model 2 | Model 3 | Model 4 | Model 5 | Model 6 |
|  | AME/SE | AME/SE | AME/SE | AME/SE | AME/SE | AME/SE | AME/SE | AME/SE | AME/SE | AME/SE | AME/SE | AME/SE |
| Less than HS | 0.0023 |  |  | 0.0019 |  |  | 0.0036 |  |  | 0.0109 |  |  |
|  | (0.0024) |  |  | (0.0024) |  |  | (0.0104) |  |  | (0.0114) |  |  |
| HS grad | 0.0006 |  |  | -0.0024+ |  |  | -0.0025 |  |  | 0.0039 |  |  |
|  | (0.0014) |  |  | (0.0013) |  |  | (0.0073) |  |  | (0.0064) |  |  |
| Some college | -0.0008 |  |  | -0.0018+ |  |  | -0.0012 |  |  | 0.0019 |  |  |
|  | (0.0012) |  |  | (0.0011) |  |  | (0.0067) |  |  | (0.0062) |  |  |
| College+ | 0.0018 |  |  | -0.0020+ |  |  | -0.0061 |  |  | 0.0065 |  |  |
|  | (0.0012) |  |  | (0.0011) |  |  | (0.0079) |  |  | (0.0074) |  |  |
| Men |  | 0.0013 |  |  | -0.0001 |  |  | -0.0021 |  |  | 0.0037 |  |
|  |  | (0.0012) |  |  | (0.0011) |  |  | (0.0060) |  |  | (0.0058) |  |
| Women |  | 0.0002 |  |  | -0.0029** |  |  | -0.0020 |  |  | 0.0056 |  |
|  |  | (0.0012) |  |  | (0.0011) |  |  | (0.0066) |  |  | (0.0060) |  |
| Non-smoker |  |  | 0.0008 |  |  | -0.0035*** |  |  | 0.0001 |  |  | 0.0049 |
|  |  |  | (0.0011) |  |  | (0.0011) |  |  | (0.0065) |  |  | (0.0059) |
| Current smoker |  |  | 0.0012 |  |  | -0.0004 |  |  | -0.0033 |  |  | 0.0074 |
|  |  |  | (0.0019) |  |  | (0.0017) |  |  | (0.0086) |  |  | (0.0089) |
| Former smoker |  |  | 0.0004 |  |  | 0.0001 |  |  | -0.0039 |  |  | 0.0032 |
|  |  |  | (0.0013) |  |  | (0.0012) |  |  | (0.0068) |  |  | (0.0063) |
|  |  |  |  |  |  |  |  |  |  |  |  |  |
| n | 184,203 | 184,203 | 184,203 | 184,203 | 184,203 | 184,203 | 18,427 | 18,427 | 18,427 | 18,427 | 18,427 | 18,427 |
